# Population structure and diversity of *Plasmodium falciparum* in children with asymptomatic malaria living in different ecological zones of Ghana

**DOI:** 10.1186/s12879-021-06120-9

**Published:** 2021-05-13

**Authors:** Linda Eva Amoah, Zakaria Abukari, Maame Esi Dawson-Amoah, Cheikh Cambel Dieng, Eugenia Lo, Yaw Asare Afrane

**Affiliations:** 1 Department of Immunology, Noguchi Memorial Institute for Medical Research, University of Ghana, Accra, Ghana; 2 West Africa Center for Cell Biology of Infectious Pathogens, University of Ghana, Accra, Ghana; 3 Department of Biochemistry and Biotechnology, Kwame Nkrumah University of Science and Technology, Kumasi, Ghana; 4 Department of Medical Microbiology, University of Ghana Medical School, University of Ghana, Accra, Ghana; 5 Department of Biological Sciences, University of North Carolina, Charlotte, NC 28223 USA

**Keywords:** Asymptomatic infections, Microsatellite analysis, Ecological zones, Ghana, *Plasmodium falciparum*, Population structure, Genetic diversity

## Abstract

**Background:**

Genetic diversity in *Plasmodium falciparum* populations can be used to describe the resilience and spatial distribution of the parasite in the midst of intensified intervention efforts. This study used microsatellite analysis to evaluate the genetic diversity and population dynamics of *P. falciparum* parasites circulating in three ecological zones of Ghana.

**Methods:**

A total of 1168 afebrile children aged between 3 to 13 years were recruited from five (5) Primary schools in 3 different ecological zones (Sahel (Tamale and Kumbungu), Forest (Konongo) and Coastal (Ada and Dodowa)) of Ghana. Asymptomatic malaria parasite carriage was determined using microscopy and PCR, whilst fragment analysis of 6 microsatellite loci was used to determine the diversity and population structure of *P. falciparum* parasites.

**Results:**

Out of the 1168 samples examined, 16.1 and 39.5% tested positive for *P. falciparum* by microscopy and nested PCR respectively. The genetic diversity of parasites in the 3 ecological zones was generally high, with an average heterozygosity (*He*) of 0.804, 0.787 and 0.608 the rainy (peak) season for the Sahel, Forest and Coastal zones respectively. The mean *He* for the dry (off-peak) season were 0.562, 0.693 and 0.610 for the Sahel, Forest and Coastal zones respectively. Parasites from the Forest zone were more closely related to those from the Sahel than from the Coastal zone, despite the Coastal zone being closer in physical distance to the Forest zone. The fixation indexes among study sites ranged from 0.049 to 0.112 during the rainy season and 0.112 to 0.348 during the dry season.

**Conclusion:**

A large asymptomatic parasite reservoir was found in the school children during both rainy and dry seasons, especially those in the Forest and Sahel savannah zones where parasites were also found to be related compared to those from the Coastal zone. Further studies are recommended to understand why despite the roll out of several malaria interventions in Ghana, high transmission still persist.

**Supplementary Information:**

The online version contains supplementary material available at 10.1186/s12879-021-06120-9.

## Introduction

Ghana comprises of three distinct ecological zones; Sahel zone (Northern Ghana), the Forest zone (Middle belt) and the Coastal zone (Southern Ghana). The prevalence of malaria varies across the three ecological zones, with the Forest zone having relatively higher parasite prevalence (22.8%) [1] compared to other zones [2]. Parasite prevalence peaks during the single rainy season (June–October) in the Sahel zone. However, malaria parasite prevalence peaks twice (May–June and October–November peaks) in a year and coincides with the bi-modal rainfall pattern in both the Forest and Coastal zones in Ghana [3, 4].

Genetic diversity in *P. falciparum* parasites primarily results from recombination between different clones in addition to within clone polymorphisms including chromosomal deletions, gene duplication, number of repeat sequences and point mutations at various genetic loci [5]. Information on parasite diversity and population structure are highly relevant to the epidemiology of malaria and virulence of the parasite [6]. The population structure of the parasite can help to determine the variations in malaria transmission between the different ecological zones as well as within the same ecological zone at different time points [7] .

Microsatellite markers are simple sequence repeats found in the parasites genome that have proven to be selectively neutral except in instances such as when they are found near genes which confer drug resistance. These markers are extremely abundant in the genome of *P. falciparum* [8] and vital in determining diversity and distribution of various parasites genotypes [9] across different transmission settings [9, 10]. The number of microsatellite markers that have been used to determine the diversity and population structure of *P. falciparum* ranges from six [11] to as high as 26 [12]. A complexity of the diversity of a parasite generally increases with the number of markers used in the analysis, however, some markers are more polymorphic than others, as such the use of a select few markers that have very high diversity can produce similar complexity as the use of a larger number of markers that have low to moderate diversity.

Many studies have shown reduced genetic diversity of the parasite populations as the result of intensified malaria control measures [13, 14]. However, the use of certain anti-malarial drugs can also alter the genetic landscape of the parasite and how they spread in a specific geographic region as a consequence of the effect of selection in favor of certain genotypes and or phenotypes [15].

Asymptomatic malaria infections present an opportunity for the mosquito vector to obtain a continuous source of parasites, which are subsequently transmitted to a new human host. The continued inoculation of genetically diverse malaria parasites into a host by different mosquitos can result in the generation of highly diverse parasites due to outcrossing and recombination events within the mosquito midgut when gametocytes of these genetically diverse parasites are picked up together by a feeding mosquito [16]. This study identified the prevalence of asymptomatic *P. falciparum* carriage by school children without any outward symptom of malaria living in five communities across three ecological zones of Ghana and evaluated the genetic diversity and population structure of the identified *P. falciparum* parasites.

## Materials and methods

### Study area

**Fig. 1:**
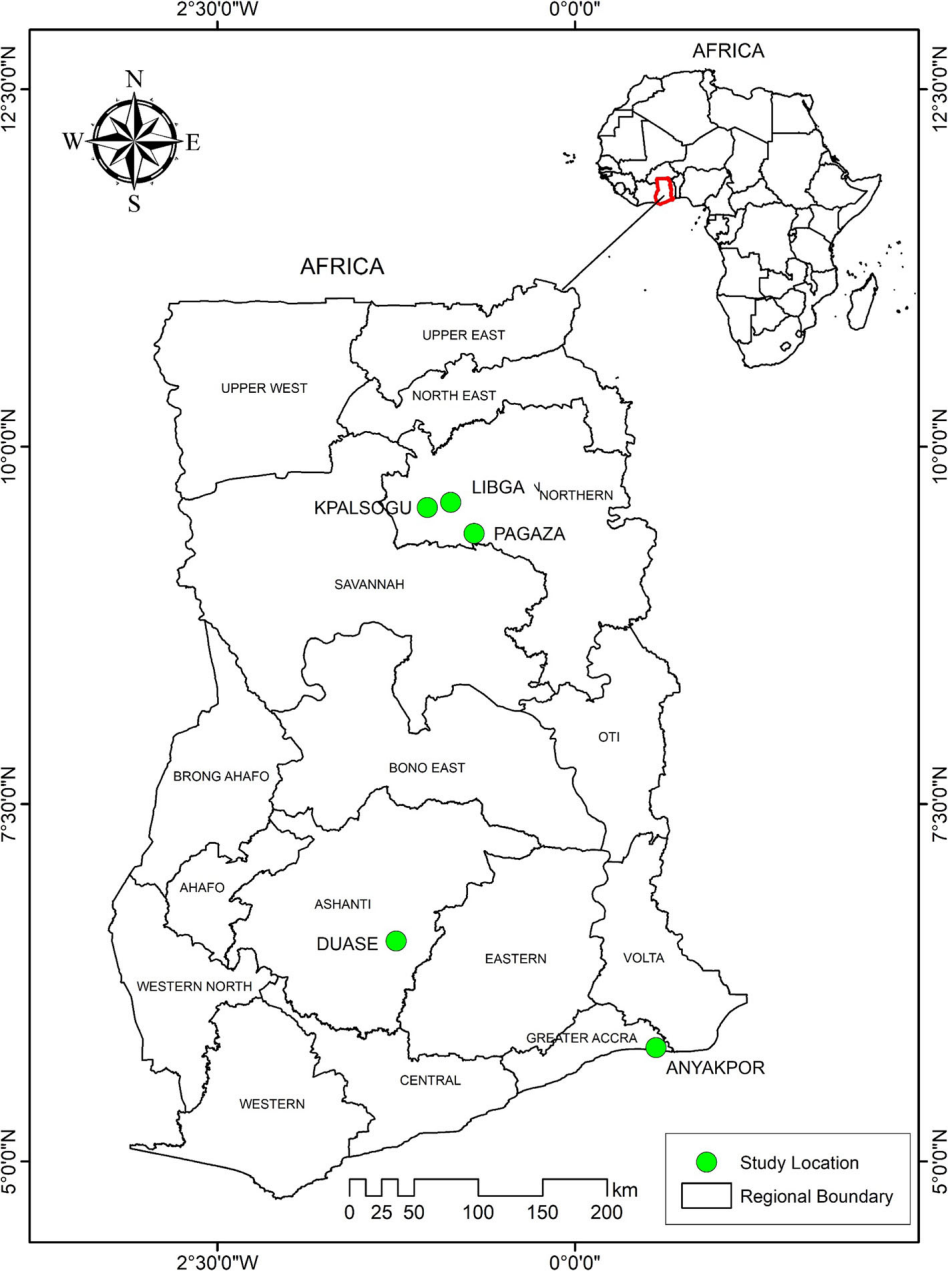
Map of Ghana highlighting the study sites. The study sites are represented by green squares on the map. The map was created for this study by Mr. Richard Adade, GIS & Remote Sensing Unit, Department of Fisheries, using shapefiles from the Survey Department of the Ghana Statistical Services and ArcMap GIS v10.5. No administrative permission was required to use the shape files. The shapefiles can be published under a CC-BY 4.0 license

The study was carried out on samples collected from five study sites in three ecological zones. These sites were Ada (5°47′00.0″N 0°38′00.0″E) and Dodowa (5°53′00.0″N 0°07′00.0″E) in the Coastal zone, Konongo (06°37′00″N 01°13′00″W) in the Forest zone and Tamale (09°24′27″N 00°51′12″W) and Kumbungu (09°24′27″N 00°51′12″W) in the Sahel zone (Fig. 1).

The Coastal zone in the south of Ghana and the Forest zone in the middle of Ghana, have two rainy seasons, a long one from April to June and the short in September to October. Average temperature is between 23 °C and 28 °C throughout the year and maximum temperatures reaching 33 °C. The peaks of malaria prevalence usually lag 1 to 2 months after the rains. Malaria vectors in the area are *Anopheles gambiae* sensu stricto and *A. funestus* [17, 18]*.*

*The Sahel zone in the north of Ghana has a unimodal rainy season from May to November. Malaria transmission follows the same pattern.* The mean annual temperature is 28 °C which can get to a maximum of 42 °C. Malaria vectors in the area are *Anopheles gambiae* s s, *An. arabiensis* and *A. funestus* [19]*.*

### Study design and population

Two cross-sectional studies were conducted in March (dry/off-peak season) and July (rainy/peak season) of 2017 in all the sites, except for in Dodowa, where samples were only collected in July. A total of 1168 afebrile children aged between 3 and 13 years attending primary schools within the study sites were recruited. All children within the stated age group, regardless of sex and socio-economic status were eligible but only those whose parent/guardian provided written parental consent and assent were enrolled.

### Ethics approval

The study was performed in accordance with the Declaration of Helsinki. Ethical approval (CHS-Et/M.5 – P1.9/2017–2018) was obtained from the College of Health Sciences Ethical and Protocol Review Committee (EPRC) of the University of Ghana. Written parental consent was obtained from parents or guardians for all the children recruited in this study. All children aged 12 years old and above were also made to endorse a child assent form. All methods were carried out in accordance with relevant guidelines and regulations.

### Sample collection

Finger-prick blood (~ 100 μl) was collected from each child and used to prepare thick and thin blood smears on microscope slides according to previously described protocols [20, 21]. Dried blood spots were also prepared by dropping 50 μl of blood onto strips of Whatman™ #3 filter paper. The thick and thin smears were processed and stained with Giemsa according to previously published protocols [22, 23]. Parasites in the thick smears were counted against 200 leukocytes when the slide was positive; otherwise, the whole slide was carefully scanned before being declared negative. Parasite densities were converted to number of parasites per microliter of blood, assuming a leukocyte count of 8000 cells/μl [24]. The blood spots (DBS) were air-dried and each placed into a zip lockä bag containing silica gel [25]. Parasites in the thick smears were counted against 200 leukocytes when the slide was positive; otherwise, the whole slide was carefully scanned before being declared negative. Parasite densities were converted to number of parasites per microliter of blood, assuming a leukocyte count of 8000 cells/μl [24]. The blood spots (DBS) were air-dried and each placed into a zip lock bag containing silica gel [25].

### Genomic DNA extraction and molecular characterization

Genomic DNA was extracted from each of the DBS using the Chelex extraction method [25, 26]. *Plasmodium falciparum* was identified using a species-specific nested PCR protocol that targeted the 18S rRNA gene [25, 27–29].

The hemi nested PCR protocol and subsequent automated capillary electrophoresis used in this study has been previously described [29]. Six microsatellite markers (Poly_α, TA40, ARA2, TA87, TAA81, and PfPK2) previously identified as being the most polymorphic of 12 markers used to genotype *P. falciparum* circulating in southern Ghana [29] were selected for use in this study. Genomic DNA from total of 119 randomly selected *P. falciparum* positive samples (40 from Ada representing the Coastal zone), 37 from Tamale (representing the Sahel zone) and 42 from Konongo (representing the Forest zone)) collected during the rainy and dry seasons were used. Every PCR plate was set up to include a negative control (no DNA template) and positive control (gDNA from the 3D7 strain of *P. falciparum* (MRA-102A) [9, 30]. Capillary electrophoresis was performed using the Applied Biosystems (ABI) 3130 series genetic analyzer (Applied Biosystems, USA). The chromosomal location and other details of the primers used are in S1 Table 1.

**Table 1:** Demographic characteristics of our study population

Coastal					Forest		Sahel		
	Ada		Dodowa	Konongo		Kumbungu		Tamale	
	Dry (116)	Rainy (148)	Rainy (59)	Dry (117)	Rainy (216)	Dry (85)	Rainy (132)	Dry (103)	Rainy (192)
Male (N (%))	42 (36.2)	58 (39.2)	14 (23.7)	60 (51.3)	116 (53.7)	28 (32.9)	63 (47.7)	59 (57.3)	109 (56.8)
Age/yrs									
Mean (SEM)	9.0 (0.21)	9.3 (0.17)	9.6 (0.23)	8.9 (0.23)	9.3 (0.13)	9.6 (0.19)	9.3 (0.21)	9.2 (0.21)	9.2 (0.15)
Min - Max	3–13	4–12	4–12	3–12	3–13	5–13	3–13	4–12	4–13
PD (p/μl)									
Mean (SEM)	53.5 (13.95)	84.9 (20.72)	120.6 (35.69)	38.0 (2.8)	82.4 (14.7)	69.0 (14.36)	75.8 (21.09)	40.2 (6.06)	230.4 (105.4)
Min - Max	18–229	8–401	24–379	22–61	6–549	33–132	14–453	16–111	14–3254
Micro (N (%))	14 (12.1)	19 (12.8)	10 (16.9)	15 (12.8)	52 (24.1)	6 (7.1)	24 (18.2)	14 (13.6)	34 (17.7)
PCR (N (%))	24 (20.7)	26 (17.6)	18 (30.5)	70 (59.8)	127 (58.8)	21 (24.7)	65 (49.2)	29 (28.2)	81 (42.4)

*N* Count; *SEM* Standard Error of Mean; *Min* Minimum; *Max* Maximum; *Pos* Positive; *PD* Parasite density is reported as parasites (p)/μl; *Micro* Parasite prevalence estimated by microscopy; *PCR* Parasite prevalence estimated by PCR

### Data analysis

GraphPad Prism v5 was used to determine the descriptive statistics including the mean and standard error of the mean as well as Kruskal Wallis test and Dunn’s multiple comparison (Post hoc test) where necessary. Pearson Chi square test (IBM SPSS v22) was used to determine variations in parasite prevalence by both microscopy and PCR between the different sites and seasons.

### Microsatellite analysis

The length of PCR products was determined with reference to an internal size standard (LIZ 600) using the GeneMapper software v5 (Applied Biosystems, USA)**.** Alleles were scored manually using Peak Scanner Software (Applied Biosystems, USA) a height of 100 relative fluorescence units (rfu) used as the minimal peak threshold, any allele with a peak value of at least 100 rfu was scored. The size range of the alleles and the average number of alleles per loci for each population were calculated [31]. The allele sizes obtained from the control *P. falciparum* strain, 3D7 were used to correct run-to-run variation among capillary electrophoresis runs.

### Genetic diversity, multiplicity of infections, and linkage disequilibrium

Results obtained after the capillary electrophoresis was analyzed using the genetic analysis software GeneAlEx [32] and was used to determine expected heterozygosity *He*, allele frequency and allelic ranges. The expected heterozygosity (*He*) was measured at each locus in each of the study sites as (*He* = [n/ (n-1)] [1-Σpi^2^]), where n is the number of isolates and pi is the frequency of the i*th* allele.

Multi locus linkage disequilibrium (LD) was assessed from each of the sites using LIAN 3.5 [33]. The standardized index of association (*I*_A_^S^), which quantitatively measures the strength of LD ranges from 0 (lack of LD in the loci) as seen in most high parasites locations to 1 (strong LD in loci) as in low transmission settings [30, 34] and is viewed as a function of the rate of recombination among samples, was calculated using the Monte Carlo method that tests the significant level of *I*_*A*_^*s*^ values [35] at 10,000 random permutations of the data [35]. at 10,000 random permutations of the data.

Among all 119 samples, 107 were polyclonal infections and 83 of these samples showed > 1 allele in two or more loci (Table 1). Because we were unable to confidently differentiate the haplotypes of the different clones, analyses were performed with two datasets: (1) all 119 samples of which only the predominant haplotypes were used in multi-clonal samples; (2) 60 single-clone haplotypes identified from 12 monoclonal (12 single-clone haplotypes) and 24 biclonal (48 single-clone haplotypes) samples of which haplotypes were unambiguously differentiated (Table 1).

Multiplicity of infection (MOI) refers to the number of distinct parasites clones in a single infection and was determined using the ratio of the total number of distinct parasites clones for a gene to the number of samples positive for the same gene. *Plasmodium falciparum* infections were classified as monoclonal if it contained only one parasite clone (one allele at all of the 6 loci) or polyclonal if the infection contained multiple alleles at any of the 6 loci [36].

### Genetic clustering analyses

An *F*_ST_ analysis was conducted using θ, an *F*_ST_-estimator in SPAGeDi v1.2e [37]. The *F*_ST_ values were tested for significance using 10,000 permutations. The pair Wright’s Fixation index (*F*_ST_) was used to determine the population structure with *F*_ST_ value ranged from 0 to 0.05 indicates low genetic variability, 0.05–0.15 indicates moderate genetic variability, 0.15–0.25 indicated great genetic differentiation and > 0.25 indicates substantial genetic differentiation [38].

A model-based Bayesian method implemented in STRUCTURE v2.3.4 was performed to examine partitioning of individuals to genetic clusters [39]. The number of clusters (*K*) was determined by simulating a range of *K* values from 1 (no genetic differentiation among all sites) to 5 (all sites were genetically differentiated from one another). The posterior probability of each value was then used to detect the modal value of Δ*K*, a quantity related to the second order rate of change with respect to *K* of the likelihood function [40]. Posterior probability values were estimated using a Markov Chain Monte Carlo (MCMC) method. A burn-in period of 500,000 iterations followed by 10^6^ iterations of each chain was performed to ensure convergence of the MCMC. Each MCMC chain for each value of *K* was run ten times with the independent allele frequency option that allows individuals with ancestries in more than one group to be assigned into one cluster. Individuals were assigned into *K* clusters according to membership coefficient values (Q) ranged from 0 (lowest affinity to a cluster) to 1 (highest affinity to a cluster). The partitioning of clusters was visualized with DISTRUCT [41]. Neighboring-joining trees were constructed using T-REX [42] to show the genetic relatedness among *P. falciparum* clones at two different levels: (i) within polyclonal individuals where only individuals with multiple alleles detected in a single locus were included and differentiated into separate clonal genotypes; (ii) among monoclonal samples from all study sites. The squared Euclidean distance, which is based on the number of times a certain allele found in two clones [43] was calculated and used for tree constructions. The resulted trees were visualized in FigTree v1.4.2 [44].

## Results

A total of 1168 children aged between 3 and 13 years old were enrolled, of which 534 (45.7%) were males and 634 (54.3%) females. The mean ages of the children from all the five sites were similar (Kruskal wallis test, *p* = 0.718) (Table 1).

### Prevalence *of Plasmodium falciparum* in the children

The overall prevalence of samples with *P. falciparum* parasites detected by microscopy was 16.1% (188/1168). Malaria parasite carriage in the dry season was highest in Tamale (13.6%) and lowest in Kumbungu (7.1%). In the rainy season, malaria parasite carriage was highest in Konongo (24.1%) and lowest in Ada (12.8%) (Table 1). Malaria parasite prevalence in both the dry and rainy season in Ada and Tamale were similar, whilst parasite prevalence in Konongo and Kumbungu were significantly higher (Pearson Chi-square value = 5.980, *p* = 0.015 and Pearson Chi-square value = 5.369, *p* = 0.026 respectively) in the rainy season than the dry season. From a zonal perspective, parasite carriage estimated by microscopy in the children from the Coastal zone (Ada and Dodowa) were similar across the dry and rainy seasons, but parasite carriage were significantly higher (Pearson Chi-square value = 5.980, *p* = 0.009 and Pearson Chi-square value = 4.860, *p* = 0.030 respectively) in children in the Forest (Konongo) and the Sahel (Tamale and Kumbungu) zones during the rainy season than the dry season (Table 2).

**Table 2:** Zonal presentation of parasite prevalence

	Coastal		Forest		Sahel	
	Dry	Rainy	Dry	Rainy	Dry	Rainy
PCR (n/N)	24/116	44/207	70/117	127/216	50/188	146/324
%	20.7%	21.3%	59.8%	58.8%	26.6%	45.1%
Micro (n/N)	14/116	29/207	15/117	52/216	20/188	58/324
%	12.1%	14.0%	12.8%	24.1%	10.6%	17.9%

*Micro* Microscopy; *N* Total number of children tested; *n* Number of parasite positive children. The numbers in the table represent exact counts. The Coastal zone was represented by samples from only Ada in the dry season as no samples were collected in Dodowa during the dry season. Coastal zone, Ada and Dodowa; Forest zone, Konongo; Sahel zone, Kumbungu and Tamale

Molecular analysis (PCR) of parasite prevalence identified 39.5% (461/1168) of the children to be positive for *P. falciparum* parasites. This was more than twice that detected by microscopy (Table 1**)**. All microscopy positive samples were confirmed to be positive for *P. falciparum* by PCR. Children living in Konongo had the highest prevalence of PCR detectable parasites during both the dry (59.8%) and the rainy season (58.8%) (Table 1). Children living in Ada had the lowest prevalence of asymptomatic parasite carriers in both the dry 20.7% (24/116) and the rainy season 17.6% (26/148) (Table 1). Parasite prevalence identified in the dry and rainy season were similar for children from Ada and Konongo, however parasite prevalence in children from Kumbungu and Tamale was significantly higher (Pearson Chi-square value = 13.011, *p* < 0.001 and Pearson Chi-square value = 5.805, *p* = 0.017 respectively) in the rainy season than in the dry season (Table 2).

### *Plasmodium falciparum* genetic diversity

**Fig. 2:**
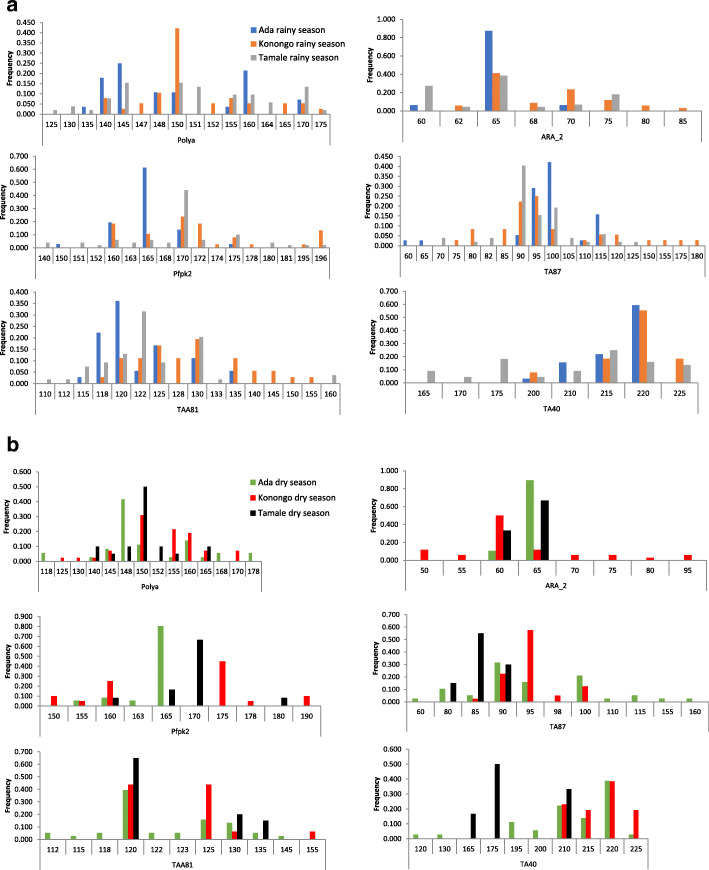
Allelic diversity at the 6 microsatellite loci. Alleles (vertical axis) were scored by GeneMapper v5 and GenAIEx and used to generate allelic frequencies of parasites collected in: (**a**) rainy season from Tamale in the Sahel zone (grey bars), Konongo in the Forest zone (Yellow bars) and Ada in the Coastal zone (blue bars) and (**b**) in the dry season from Tamale in the Sahel zone (black bars), Konongo in the Forest zone (red bars) and Ada in the Coastal zone (green blue bars)

A total 119 parasites were genotyped at six polymorphic loci (Fig. 2, S2a Table and S2b Table). These 6 microsatellite loci were chosen because they identified the highest level of polyclonality in a sample in a previous study [29]. There were 40 samples from the Coastal zone (Ada), 42 from the Forest zone (Konongo) and 37 from the Sahel zone (Tamale). Overall, the most polymorphic locus was the Pfpk2 with 14 distinct alleles at Tamale and the least polymorphic locus was ARA2 with only 4 distinct alleles at Ada in the rainy season. In the dry season the most polymorphic locus was TAA81 at Ada with 10 distinct alleles while ARA2 recorded only two distinct alleles for both Ada and Tamale.

Allelic variation patterns in each of the six MS loci genotyped in the rainy season were not significant, allelic size variation were between 1 and 10 bp except TA40 locus which recorded allelic variation of 25 bp between 2 alleles in the marker (Fig. 2a). In the dry season, allelic variations patterns were similar to that in the rainy season except TA40 which recorded allelic size variation of 35 bp of 2 alleles at this locus (Fig. 2b and S2 Table 2). The allelic variations that were observed in each of the 6 loci genotyped in both rainy and dry seasons across the 3 ecological zones were not significantly different (Kruskal Wallis test, *P* = 0.9630).

In the rainy season, Poly_α locus had the highest *He* (0.886) and ARA2 the lowest *He* (0.735), average *He* = 0.804 in the Sahel zone (Tamale). In the Coastal zone (Ada), Poly_α similarly had the highest *He* (0.829) and ARA2 the lowest He (0.227), average *He* = 0.608. However, parasites from the Forest zone (Konongo) showed the highest diversity at the TAA81, *He* = (0.877) and TA40 the lowest *He* (0.602), Average *He* = 0.787 (S2 Table 2). In the dry season, Poly_α had the highest *He* (0.705) and ARA2 the least *He* (0.444) at Sahel zone (Tamale) with average *He* of 0.562. In the Coastal zone (Ada), TA87 had the highest *He* (0.812) and Pfpk2 had the least *He* (0.338) with average *He* of 0.610. In the forest zone (Konongo) Poly_α locus had the highest *He* (0.805) while TA87 had the least *He* (0.600) with an average *He* of 0.693 (S2 Table 2). The overall heterozygosity values (0.61 ± 0.09, 0.78 ± 0.04 and 0.80 ± 0.02) for Ada, Konongo and Tamale respectively in the rainy season were not higher than those (0.61 ± 0.11, 0.69 ± 0.03, and 0.56 ± 0.03) for the same sites in the dry season (S2 Table 2).

### Multiplicity of parasites infection

Samples from the Forest zone (Konongo) had the highest number of different parasite clones, with MOI range of 2.6 (TAA81) to 1.3 (TA40) and a mean MOI of 2.0. Multiplicity of infection in the Coastal zone (Ada) ranged between 1.8 (TA87) and 1.1 (ARA2) with a mean MOI of 1.4. The average MOI in samples from the Sahel zone (Tamale) was similar to that reported from samples collected from the Forest zone (Konongo), MOI range from 2.3 (Poly_α) to 1.5 (ARA2), mean MOI was 1.7 in the rainy season. In the dry season, MOI in the Coastal zone (Ada) ranged from 1.6 (Poly_α) to 1.0 ARA2, mean MOI was 1.4 compared to MOI of 1.3 (Poly_α) to 1.0 (TA40) with mean MOI of 1.1 for the Sahel zone (Tamale) and 2.4 (Poly_α) to 1.0 (TAA81) with mean MOI of 1.4 for the Forest zone (Konongo). The average MOI of asymptomatic infections of *P. falciparum* in the Forest and Sahel zones (Konongo and Tamale) were higher than those from the Coastal zone (Ada).

Among the 119 *P. falciparum* infections 12 (10.1%) of the infections were monoclonal and 107 (89.9%) were biclonal and polyclonal across the three ecological zones (Table 3). The genotypes of these samples were differentiated into separate clones in the analysis.

**Table 3:** Sample size and distribution of monoclonal, biclonal, and polyclonal samples from each of the study sites and collection seasons. Asterisk denotes samples with more than 2 alleles detected at two and more genetic loci

Study site	Collection season	Sample size	Monoclonal (%)	Biclonal (%)	Polyclonal ^a^ (%)
Ada (Coastal)	Rainy	19	0	2 (10.5%)	17 (89.5%)
	Dry	21	6 (28.6%)	2 (13.3%)	13 (61.9%)
Konogo (Forest)	Rainy	20	1 (5%)	0	19 (95%)
	Dry	22	0	12 (54.5%)	10 (45.5%)
Tamale (Sahel)	Rainy	27	2 (7.4%)	3 (11.1%)	22 (81.5%)
	Dry	10	3 (30%)	5 (50%)	2 (20%)
	**Total**	**119**	**12 (10.1%)**	**24 (20.2%)**	**83 (69.7%)**

^a^ Samples with > 2 alleles detected at two and more genetic loci

### Linkage disequilibrium

In order to assess the non-random association of *P. falciparum* microsatellites haplotypes thus Linkage disequilibrium (LD) in the 6 MS loci genotyped the Monte Carlo simulation model was used [35]. Based on the LIAN analyses of all 119 samples, *I*_A_^S^ values ranged from 0.036 in the Konongo (Forest zone) to 0.047 in Tamale (Sahel zone; Table 4). When all sites were pooled together, LD was observed with high levels of significance (*I*_A_^S^ = 0.027, *P <* 0.01; Table 4). For the 60 single-clonal haplotypes (12 monoclonal and 24 biclonal samples; Table 3), *I*_A_^S^ values ranged from 0.062 in Konongo (Forest zone) to 0.140 in Ada (Coastal zone; Table 4). Linkage disequilibrium was also observed with high levels of significance when all sites were pooled together, (*I*_A_^S^ = 0.058, *P <* 0.01; Table 4).

**Table 4:** Linkage disequilibrium statistics based on LIAN analyses with all 119 samples and 60 single-clonal haplotypes, respectively

Region (*n* = 119)	*I*_A_^S^	*P*-value
Ada (Coastal)	0.044*	< 1.00 × 10^− 02^
Konongo (Forest)	0.036*	< 1.00 × 10^− 02^
Tamale (Sahel)	0.047*	< 1.00 × 10^− 02^
All	0.027*	< 1.00 × 10^− 02^
Region (*n* = 60)	*I*_A_^S^	*P*-value
Ada (Coastal)	0.140*	< 1.00 × 10^− 02^
Konongo (Forest)	0.062*	< 1.00 × 10^− 02^
Tamale (Sahel)	0.113*	< 1.00 × 10^− 02^
All	0.058*	< 1.00 × 10^− 02^

* *P* < 0.01; Ada (Coastal zone), Konongo (Forest zone), Tamale (Sahel zone)

### Genetic relatedness of *P. falciparum* parasites

**Fig. 3:**
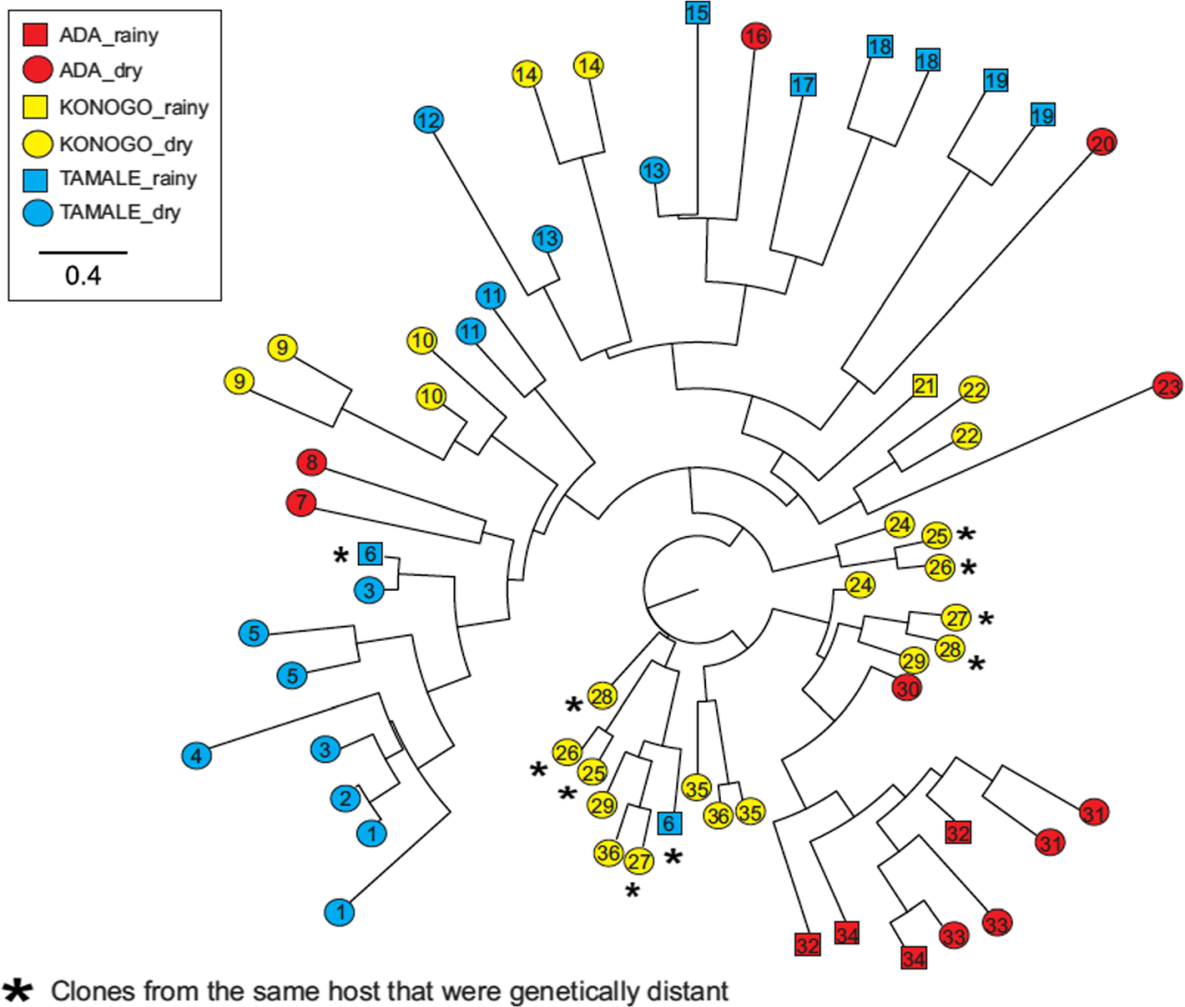
Intra-host diversity predicted by microsatellite markers among 48 single clones identified from 26 samples. Samples were collected from Tamale in the Sahel zone (red), Konongo in the Forest zone (yellow), and Ada in the Coastal zone (blue). Samples from the rainy season are represented by squares, whilst samples from the dry season are represented by circles. Clones identified from the same individual were indicated by the same number. Clones in samples from six hosts that were shown to be genetically distant from each other were labeled by asterisks

Two analyses were performed to examine the genetic relatedness of *P. falciparum* samples. In the first analysis only samples that were either mono or bi-clonal infections were included in the analysis, samples with more than two clones were excluded. A total of 60 clones identified from 36 samples (12 monoclonal and 24 biclonal samples; Table 3) were included in this analysis. All the 13 samples in the Forest zone had clones detected within a host, which were genetically similar to one another, consistent with the LD results that showed a higher level of linkage/recombination in these samples (Table 4). Parasite clones identified from five of the 13 samples in Konongo (Forest zone; sample #9, 10, 14, 21, and 22 in Fig. 3) were nested within the clade that contained most samples from Tamale (Sahel Zone), indicating that these clones were closely related. Among all samples from Konongo (Forest zone), four exhibited clones that were genetically different from each other (sample #25, 26, 27, and 28 in Fig. 3; marked in asterisks). Clones from six other Konongo samples (#24, 27, 28, 29, 35, and 36) were closely related to samples from Ada (Coastal zone; sample #30–34 in Fig. 3).

**Fig. 4:**
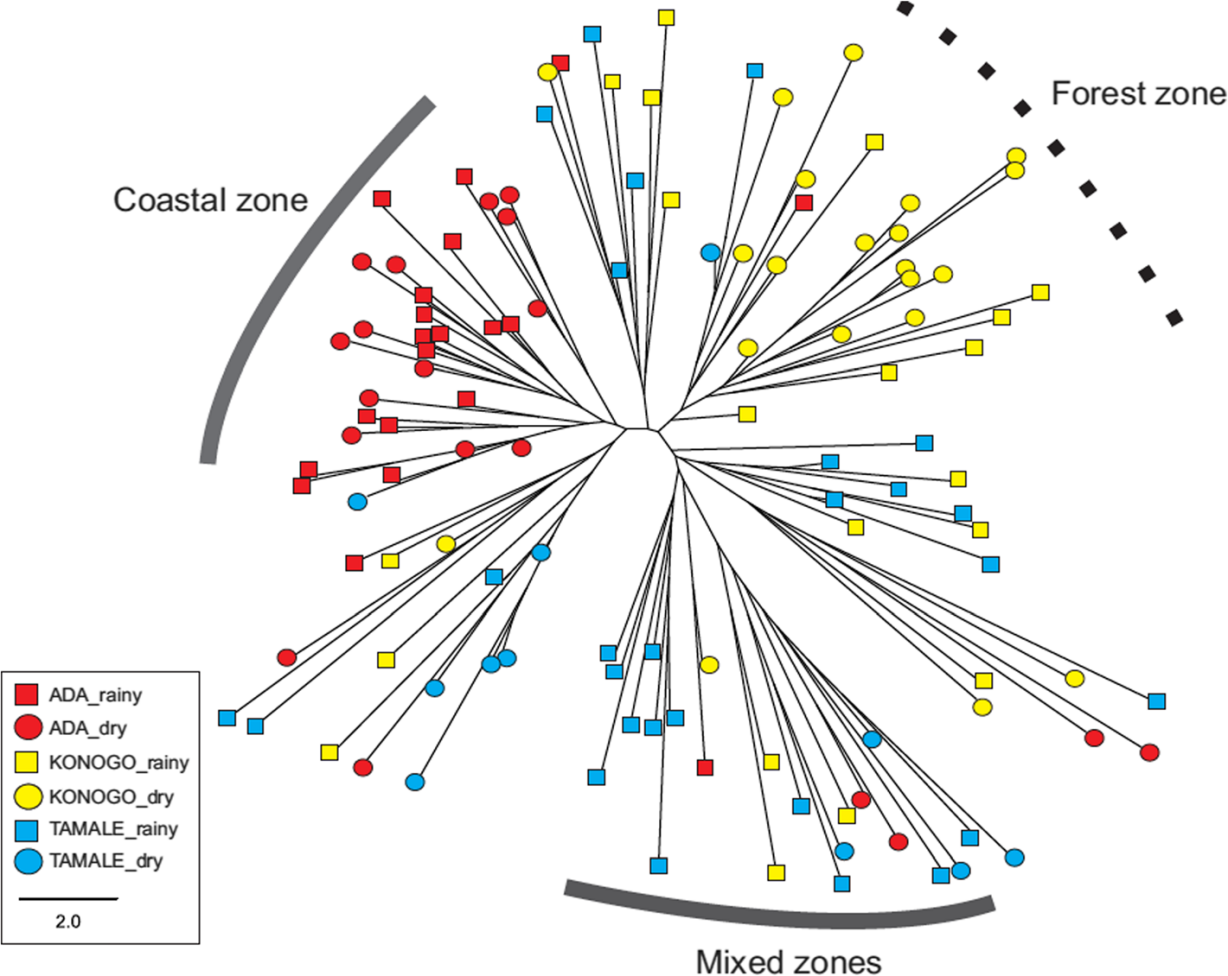
Phylogeny of the 119 *P. falciparum* samples showing genetic relatedness. Samples were collected from Tamale in the Sahel zone (light blue), Konongo in the Forest zone (yellow), and Ada in the Coastal zone (red)

In the second analysis, a phylogeny was composed based on the presence or absence of an allele, which does not show the genetic identity of individual clones. A total of 119 samples, including monoclonal and polyclonal samples were used in this analysis. Four major clades were observed in the phylogeny (Fig. 4). Two of the clades contained mostly samples from the Sahel and Forest zones, indicating a high level of genetic relatedness as shown in the single-clone phylogeny (Fig. 4). One clade had samples from the Sahel zone clustered with samples from the Coastal zone, and another clade had samples from all three ecological zones mixed together (Fig. 4).

### Population genetic structure of *P. falciparum* parasites

The pairwise genetic differentiation (FST) values were used to estimate the population structure of the six loci across the three ecological zones. The greatest differentiation was observed between Tamale (Sahel zone) and Ada (Coastal zone) (*F*_ST_ = 0.116; *P* < 0.05; Table 5) followed by Konongo (Forest zone) and Ada (Coastal zone) (*F*_ST_ = 0.095; P < 0.05; Table 5). By contrast, non-significant differentiation was observed between Tamale (Sahel zone) and Konongo (Forest zone) (*F*_ST_ = 0.035; *P* > 0.05; Table 5).

**Table 5:** Genetic Differentiation among Parasite Populations

*F*_ST_/*P*-level	Coastal	Forest	Sahel
Ada	–	**	**
Konongo	0.095	–	ns
Tamale	0.116	0.035	–

** *P* < 0.01; ns: not significant. Ada (Coastal zone); Konongo (Forest zone); Tamale (Sahel zone)

**Fig. 5:**
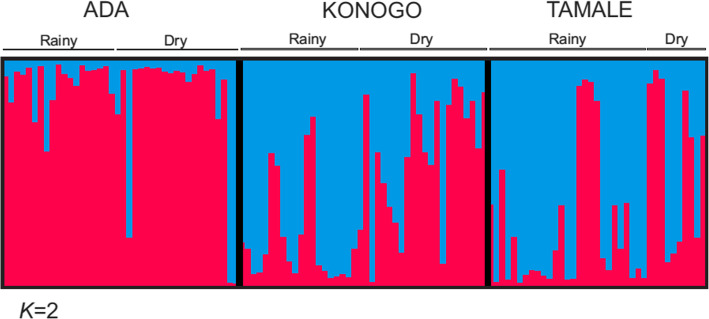
Genotype structure of parasite isolates. A Bayesian bar plot showing parasite genotype structure among samples from Tamale (Sahel zone), Konongo (Forest zone) and Ada (Coastal zone) of Ghana. Two most probable genetic clusters (red and blue) were determined. Each column represents a single sample and the color distribution in each column represents the proportion of the two clusters in each of the samples. Most of the parasite samples in the Sahel and Forest zones (Tamale and Konongo) shared the blue cluster for both rainy and dry seasons whereas parasites from the Coastal zone (Ada) had predominantly the red cluster

Two most probable genetic clusters (pink and blue) were identified by Bayesian inference for the 119 samples collected from the three zones (S3 Table 3). The majority of samples from the Sahel and Forest zones (Tamale and Konongo) shared the blue cluster whereas parasites from the Coastal zone (Ada) had mostly the pink cluster (Fig. 5), despite Coastal zone (Ada) being closer in distance to the Forest zone (Konongo) (290 km) relative to the Sahel zone (Tamale) (446 km). This clustering pattern was consistent with the level of genetic differentiation observed among regions (Table 5) and also supports the genetic relationships observed in the phylogenetic analyses (Figs. 3 & 4).

## Discussion

Asymptomatic *P. falciparum* infections are a hindrance to malaria control as they can serve as reservoirs for gametocyte carriage [45] and at times can result in symptomatic infections weeks later [46]. Investigating parasite population structure and gene flow across the three ecological zones of Ghana could help understand the impact of malaria control interventions that have been implemented in the country [47]. In this study, relatively high prevalence of asymptomatic malaria parasite carriage by the school children were recorded in the Sahel and Forest zones than the Coastal zone. The rather low prevalence of asymptomatic *P. falciparum* carriers in the Coastal zone compared to the Forest and Sahel zones might mean that ongoing malaria interventions might have worked better in the Coastal zone than the others. The higher prevalence of asymptomatic *P. falciparum* carriers in the Sahel and Forest zones is despite additional malaria control interventions such as indoor residual spraying (IRS), which is not carried out in the Coastal zone.

This high prevalence of asymptomatic malaria parasite carriage by the children in both the peak and dry malaria season may mean that additional malaria control interventions that target asymptomatic parasite carriage such as mass test and treat exercises could be implemented to reduce parasite burden [48].

The genetic diversity of the parasites identified across the three ecological zones of Ghana was generally moderate to high. In the rainy season, genetic diversity in the Sahel and Forest zones were higher than diversity in the Coastal zone (Ada), consistent with earlier findings that areas with the highest prevalence of asymptomatic parasite carriers would have the greatest genetic diversity and multiplicity of infection [49]. Parasites identified in the Sahel zone (Tamale) has the least diversity relative to parasites from the two other zones. The low heterozygosity of *P. falciparum* identified in the Coastal zone (Ada) relative to the Forest (Konongo) and Sahel zones (Tamale) could be due to relatively lower transmission intensity and consequently lower densities of mosquitoes in the Coastal zone relative to the Sahel and Forest zones (Afrane et al, unpublished). The low parasite diversity in the Coastal zone could also imply that the malaria control interventions implemented in the Coastal zone have been more effective and/or successful than in the Forest and Sahel zones as discussed already [36]. The higher parasite diversity identified in the dry season relative to the peak season in all the three zones could be the result of a higher density of major parasite clones in the peak season that prevented the detection of a number of minor parasite isolates that were present at much lower frequencies.

The level of recombination, genetic variation and genetic structure from LD and clustering analyses reflect the patterns of gene flow and transmission intensity among the parasites circulating within the three ecological zones. In areas with high transmission, the frequent recombination among parasite strains, low genetic differentiation and large parasite gene pools result in high levels of genetic variation as seen in most malaria endemic areas in sub-Saharan Africa [5, 30, 50, 51]. Similar genetic composition and phylogenetic closeness of parasites particularly between the Sahel and Forest zones suggest the likely carriage of diverse parasites by mobile population that end up mixing with the local parasite population and thereby reducing the genetic differentiation of the parasites [34]. This suggestion is supported by the fact that movement from the Sahel zone of Ghana is very high and migration from the Sahel (North) to the Coastal zone of Ghana is mainly through the Forest zone, where most of the migrant population settle and some continue their migration to the Coast [52, 53]. Similarities in the diversity of parasites from the Sahel and Forest zones relative to the Coastal zone also suggests that a recombinant vaccine based on the genetic background of a polymorphic antigen found in the Sahel zone would be equally effective in the Forest zone but may lack efficacy in the Coastal zone.

The mean MOI in samples from the Sahel zone (Tamale) was slightly lower than that identified in the Forest zone (Konongo) in the rainy season most likely due to the residents being exposed to a higher incidence of infectious mosquito bites [54], with each bite likely to inoculate a genetically diverse parasite strain. The mean MOI for the forest and coastal zones were similar (mean MOI = 1.4) except the Sahel zone that recorded mean MOI of 1.0 during the off-peak season. This supports the fact that low transmission and the dry season are associated with reduced MOI in malaria endemic countries [13, 28]. The low variations in MOI identified across the Sahel and Forest zones is contrary to an earlier report from Ghana where large differences in the MOI across different sites across the country were observed [55]. High malaria parasite prevalence settings are usually characterized by infections containing high parasite multiplicity of infection and genetic diversity [56, 57]. The multiplicity of infection identified in this study could have been higher than was reported due to the event that minor parasite clones are often undetected [58].

High parasites diversity and MOI may imply high parasite survival and successful transmission in the midst of malaria control interventions [13]. Asymptomatic cases with high MOI could progress towards symptomatic or severe form of infections. Also, genetically diverse clones may adapt better to existing interventions and increase the likelihood of developing antimalarial resistance. Most malaria control interventions are implemented without any recourse to the diversity of the parasites circulating within the implementation sites [59], however, greater success can be achieved by reducing malaria incidence as well as parasite diversity [59, 60]. A deeper investigation is needed to explore the association between polyclonality and anti-malarial drug resistance, given that malaria infections with high complexity could enhance the selection of drug resistant parasites than low complexity infections [61]. Although microsatellites analysis is a cost-effective, rapid, and user-friendly tool for determining population structure and transmission [30, 62], the genotype of each clone within a polyclonal sample cannot be easily distinguished. Amplicon deep sequencing offers an alternative means for inferring genetic relationships among clones within and between hosts [63] that can be used in future studies to thoroughly investigate the association between the complexity of infections and genetic variation in high and low transmission areas.

## Conclusion

A large asymptomatic parasite reservoir was found in the school children during both rainy and dry seasons, especially those in the Forest and Sahel savannah zones. Further studies are recommended to understand why despite the roll out of malaria interventions in Ghana, high transmission still persist. The asymptomatic *P. falciparum* parasites identified in this study had high genetic diversity and polyclonalty. Parasites from the Forest zone were more related to parasites from the Sahel zone than parasites from the Coastal zone despite the sites from the Coastal zone being closer to the Forest zone than the Sahel zone.

## Supplementary Information

**Additional file 1: Table S1.** Primers used in the study. **Table S2**. Microsatellite analysis. 2a. Genetic diversity. 2b. Complete Microsatellite data set. **Table S3**. Results of the Bayesian inference for the 119 samples collected from the three zones.

## Data Availability

All data generated or analysed during this study are included in this published article [and its supplementary information files].

## Abbreviations

MS: Microsatellites
MOI: Multiplicity of infection
CE: Capillary electrophoresis
SP: Sulphadoxine - pyrimethamine
SMC: Malaria chemoprevention
LD: Linkage disequilibrium
LSM: Larval source management
LLINs: Long lasting insecticides treated nets
*P. falciparum*: *Plasmodium falciparum*
DBS: Dried filter paper blood spots
MOI: Multiplicity of infection
FST: Pairwise genetic differentiation
LD: Linkage disequilibrium
