# Supplementary material for: Population structure and diversity of Plasmodium falciparum in children with asymptomatic malaria living in different ecological zones of Ghana
Source: BMC Infect Dis. 2021 May 13;21:439. doi: 10.1186/s12879-021-06120-9 (PMC8120845; doi:10.1186/s12879-021-06120-9)

Table s1: Primers used in the study

Locus	Primer sequence 5'-3'	Probe	Chromosome	SSR linked gene	Annealing (°C)
**Microsatellite analysis**					
**Poly α-R**	ATCAGATAATTGTTGGTA		4	DNA Poly α	42
**Poly α-F**	AAAATATAGACGAACAGA				42
**Poly α-3(IR)**	GAAATTATAACTCTACCA	6-FAM			45
**PFPK2-3R**	CCTCAGACTGAAATGCAT		12	Protein Kinase	42
**PFPK2-F**	CTTTCATCGATACTACGA				42
**PFPK2-R**	AAAGAAGGAACAAGCAGA	HEX			45
**TA81-3F**	GAAGAAATAAGGGAAGGT		5		42
**TA81-R**	TTTCACACAACACAGGATT				42
**TAA81-F**	TGGACAAATGGGAAAGGATA	Atto 565			45
**ARA2-3(F)**	GTACATATGAATCACCAA		11	Asparagine Rich Protein	42
**ARA2-R**	GCTTTGAGTATTATTAATA				42
**ARA2-F**	GAATAAACAAAGTATTGCT	6-FAM			45
**TA87-3F**	ATGGGTTAAATGAGGTACA		6		42
**TA87-R**	ACATGTTCATATTACTCAC				42
**TA87-F**	AATGGCAACACCATTCAAC	HEX			45
**TA40 Rev-1**	GAAATTGGCACCACCACA		10		42
**TA40 For**	AAGGGATTGCTGCAAGGT				42
**TA40 Rev-2**	CATCAATAAAATCACTACTA	Atto 565			45
**18S rRNA gene of *P. falciparum***					
**rPLU6**	5'TTAAAATTGTTGCAGTTAAAACG3'				
**rPLU5**	5'CYTGTTGTTGCCTTAAACTTC3'				55
**rFAL1**	5'TTAAACTGGTTTGGGAAAACCAAATATATT-3'				
**rFAL2**	5'-ACACAATAGACTCAATCATGACTACCCGTC-3'				58

Table S2. Microsatellite analysis

2a. Genetic diversity

Site	Loci	N	Na	He
**Ada rainy season**	Poly_a	14	8.0	0.829
	Pfpk2	18	5.0	0.568
	TAA81	18	7.0	0.773
	ARA2	16	3.0	0.227
	TA87	19	7.0	0.709
	TA40	16	4.0	0.547
**Konong rainy season**	Poly_a	19	11.0	0.784
	Pfpk2	19	9.0	0.839
	TAA81	18	11.0	0.877
	ARA2	17	7.0	0.746
	TA87	18	13.0	0.856
	TA40	19	4.0	0.620
**Pagaza rainy season**	Poly_a	26	12.0	0.886
	Pfpk2	25	14.0	0.776

	TAA81	27	10.0	0.818
	ARA2	22	6.0	0.735
	TA87	26	11.0	0.767
	TA40	22	8.0	0.840
**Ada dry season**	Poly_a	18	10.0	0.776
	Pfpk2	18	4.0	0.338
	TAA81	19	10.0	0.787
	ARA2	19	2.0	0.188
	TA87	19	10.0	0.812
	TA40	18	8.0	0.762
**Konong dry season**	Poly_a	21	9.0	0.805
	Pfpk2	10	6.0	0.710
	TAA81	16	4.0	0.609
	ARA2	17	8.0	0.708
	TA87	20	5.0	0.600
	TA40	13	4.0	0.725
**Pagaza dry season**	Poly_a	10	7.0	0.705
	Pfpk2	6	4.0	0.514
	TAA81	10	3.0	0.515
	ARA2	9	2.0	0.444
	TA87	10	3.0	0.585
	TA40	6	3.0	0.611

2b. Complete Microsatellite data set

Sample ID	Site/Season	Poly_a	PFPK2	TAA81	ARA2	TA87	TA40
ADA 078	Ada/Rainy	145,160	170	120	65,70	100	210
ADA 113	Ada/Rainy	0	150,165	120	65	65,95	210
ADA 061	Ada/Rainy	140,150	165	0	65	95	200,220
ADA 110	Ada/Rainy	148	160,165	130	65	90,95	215,220
ADA 120	Ada/Rainy	160	165	115,118	0	95,100	220
ADA 114	Ada/Rainy	135,140	160	120,125	65	60,95	220
ADA 155	Ada/Rainy	145,160	160,165	118,125	65	95,100	220
ADA 153	Ada/Rainy	145	165,170	122	0	100,110	220
ADA 148	Ada/Rainy	150,155	160	118	65	90,95	215
ADA 126	Ada/Rainy	140,148	165	120	0	100	220
ADA 098	Ada/Rainy	145,150	165,175	120,125	60	100,115	0
ADA 009	Ada/Rainy	145	0	120,135	65	100,115	0
ADA 128	Ada/Rainy	0	165,170	120,125	65,70	95,100	0
ADA 098	Ada/Rainy	0	165	118	65	100,115	210,220
ADA 072	Ada/Rainy	140	165,160	125,130,135	65	100,115	220
ADA 077	Ada/Rainy	160	165	120	65	95,100	215
ADA 086	Ada/Rainy	0	165,170	120,125	65	100,115	220
ADA 068	Ada/Rainy	0	165	118	65	100,115	220
ADA 132	Ada/Rainy	170	165	120,130	65	95,100	215,225
ADA 085	Ada/Dry	0	165	0	65	60,95	225,220
ADA 066	Ada/Dry	148,165	165	112,120	65	90,100	220
ADA 070	Ada/Dry	0	160,165	120,130	65	155,160	120,130
ADA 090	Ada/Dry	140,155	165	0	65	90,100	210,215
ADA 113	Ada/Dry	145,148	165	125,130	65	90	210
ADA 095	Ada/Dry	148,160	165	115,118	65	90,95	220
ADA 106	Ada/Dry	145,148	165	120,125	65	90,95	215
ADA 031	Ada/Dry	160,148	160,165	112,125	65	90,100	210,220
ADA 059	Ada/Dry	145,148	165	122,125	65	95	220

ADA 063	Ada/Dry	148,150	165	118,122	65	90,100	210,220
ADA 105	Ada/Dry	148	165	120	65	100,110	220
ADA 116	Ada/Dry	148,150	160,165	120,125	65	90,95	215
ADA 119	Ada/Dry	148	0	125,145	65	100	220
ADA 112	Ada/Dry	148	0	120,130	65	90,115	0
ADA 100	Ada/Dry	148,160	0	120	65	100,115	210,220
ADA 086	Ada/Dry	0	165	120	0	0	0
ADA 088	Ada/Dry	160	165	120	0	0	0
ADA 095	Ada/Dry	168	165	130	65	90	210
ADA 119	Ada/Dry	150	165	120	60	85	200
ADA 120	Ada/Dry	118	163	135	65	80	195
ADA 105	Ada/Dry	178	155	123	60	80	195
KG 040	Konongo/Rainy	140,145,150	160	120,125,130	0	80,90,95	225
KG 062	Konongo/Rainy	148,160	160,170,175,178	145,150,155,160	65,70,75	75,80,90	220
KG 112	Konongo/Rainy	155	170	12	62	150,175,180	200
KG 060	Konongo/Rainy	150	170	120	68	155	220
KG 079	Konongo/Rainy	155	165,170	125,130,135	70	85,90	225
KG 115	Konongo/Rainy	140,148,175	170	130,135,145	65,75,85	100,115	215,220
KG 119	Konongo/Rainy	150,165,170	160,165,172	118,130	65	95	200,215,220
KG 039	Konongo/Rainy	148,150	165	135	70	90,95,120	225
KG 015	Konongo/Rainy	150,160	170,175	0	65	95	215,220
KG 099	Konongo/Rainy	150	170,174	122,130	65,70	80,90,95	220
KG 008	Konongo/Rainy	147,150,155	0	135,140	65	95,100	220
KG 068	Konongo/Rainy	150	160,170	125,130,145	65,70	85,90,115	220
KG 106	Konongo/Rainy	140,150	160,165,170	120,128,135,140	65,75,80	85,95	220
KG 108	Konongo/Rainy	147,150	175,196	122,125,135	65,68	95	0
KG 124	Konongo/Rainy	148,150	160,196	135,140,145,170	70	90	215,220
KG 104	Konongo/Rainy	150,152,160	172,196	125,128,130	75,80	90,110	220,225
KG 033	Konongo/Rainy	150,170	172,196	122,128	65,75	100,120	215,220
KG 088	Konongo/Rainy	150,151	172,196	122,128	65	95	215
KG 054	Konongo/Rainy	150,165	172,195	125,130	0	0	225
KG 115	Konongo/Rainy	0	172	0	0	0	220

KD 005	Konongo/Dry	140,152,155	0	0	50,55,60,75	85,90	220
KD 003	Konongo/Dry	150,170	0	120	60	90	210
KD 103	Konongo/Dry	150,170,175	155,178	0	60	98	225
KD 042	Konongo/Dry	155,160	175	120	50,55,60,75,80	95	220
KD 107	Konongo/Dry	160,165	150	0	0	95	0
KD 108	Konongo/Dry	155,160	175	125	65	90	215
KD 106	Konongo/Dry	145,150,170,175	160,175	125	95		0
KD 51	Konongo/Dry	150	175	0	60	95	215
KD 080	Konongo/Dry	150,160	0	0	0	0	0
KD 111	Konongo/Dry	145,150,155,160	160	120	0	95	0
KD 013	Konongo/Dry	150,155,165	175	125	60	90	210
KD 001	Konongo/Dry	150	160	120	70	90,95	0
KD 009	Konongo/Dry	150,160,165	0	125	50	95,100	0
KD 015	Konongo/Dry	150	0	125	60	100	0
KD 0711	Konongo/Dry	150,155	0	155	65	90,95	215,225
KD 068	Konongo/Dry	150,155,170	0	125	60	95	0
KD 109	Konongo/Dry	155,160	0	120	0	95	0
KD 015	Konongo/Dry	150,155,160,165	0	125	60	95	220
KD 054	Konongo/Dry	0	190	130	60,65	100	210
KD 179	Konongo/Dry	130,145,150,155	0	120	60	95	220
KD 033	Konongo/Dry	160	0	120	60	95	225
KD 088	Konongo/Dry	150,160	0	0	0	95	220
PZ 096	Tamale/Rainy	150	140	120	0	0	200
PZ 024	Tamale/Rainy	130	170	110,118	68	82	175
PZ 034	Tamale/Rainy	140,145,155,160,164	165	120,130,133	60	70	175
PZ 090	Tamale/Rainy	150,155	168	120	62	90	175
PZ 051	Tamale/Rainy	170	160	118	65	90	175
PZ 194	Tamale/Rainy	130,135	163	122	65	105	170
PZ 100	Tamale/Rainy	0	180	130	60	90	165
PZ 013	Tamale/Rainy	164	0	115	65	90	165
PZ 055	Tamale/Rainy	170,151	170,181	122,125	60,65,75	90,95,100	0
PZ 085	Tamale/Rainy	151,155,160	170,172	122,130	70,75	95,100,125	215

PZ 093	Tamale/Rainy	151	170,172	122	65	90,95	215,225
PZ 019	Tamale/Rainy	145,151,158	170	122,125	65	95	215,220
PZ 170	Tamale/Rainy	150,155	170,175	122,130,140	0	95,100	215,220
PZ 194	Tamale/Rainy	125,150	152,170	122,160	0	90,95	215
PZ 047	Tamale/Rainy	150,175	151,170	122	0	90,100	215,225
PZ 043	Tamale/Rainy	150,170	160,175	120,125,130	60	100	
PZ 104	Tamale/Rainy	140,145,152,155,160	165,170	115,125	60,65	90,115	
PZ 054	Tamale/Rainy	145,155	0	115,120,130	60,65,70	90,100	0
PZ113	Tamale/Rainy	140,145	170,175	112,118	60	100,115,120	210,220
PZ 004	Tamale/Rainy	145,160,170	170,172	122	65,75	100	215,220
PZ 028	Tamale/Rainy	145,151,165	170	125,130	75	90,100	215,225
PZ 160	Tamale/Rainy	150,160,165,170	170,175	120,130	65,70	90,95	220,225
PZ 187	Tamale/Rainy	150,170	170,175	122,130	65	90,95	
PZ 178	Tamale/Rainy	145,160	170,195	122,130	60,65,70	90,100	210,215
PZ 078	Tamale/Rainy	151,170	170,196	122	60	90,115	0
PZ 155	Tamale/Rainy	140,170	170	118,122	75	90,110	210,225
PZ 048	Tamale/Rainy	151,170	151,170	122,130	0	80,90,95	210,225
PAG 027	Tamale/Dry	150	0	120	65	85,90	0
PAG 087	Tamale/Dry	150	0	120	65	85	210
PAG 014	Tamale/Dry	148	0	120	65	80,85,90	210
PAG 057	Tamale/Dry	150,165	170	135	60	85	175
PAG 050	Tamale/Dry	152	160,180	120	65	90	175
PAG 048	Tamale/Dry	150,165	0	130	0	90	0
PAG 051	Tamale/Dry	145,155	170	120	60	85	0
PAG 020	Tamale/Dry	140	170	120	65	85	175
PAG 043	Tamale/Dry	150	165	130	60	80	165
PAG 026	Tamale/Dry	150	170	120,135	65	85	0

S3. Results of the Bayesian inference for the 119 samples collected from the three zones

# of cluster	1	2	3	4	5
	-2500.9	-2317.2	-2270.8	-2201.8	-2197.7
	-2501.5	-2317.6	-2267.7	-2201.9	-2201.5
	-2500.5	-2317.4	-2270.3	-2201.1	-2200.8
	-2501.3	-2317.3	-2267.2	-2202.3	-2202.9
	-2501	-2317.6	-2270.1	-2200.8	-2199.2
	-2501.1	-2317.6	-2266	-2202	-2213.7
	-2501.3	-2317.3	-2272	-2201.1	-2203
	-2501	-2317.7	-2268.8	-2200.7	-2195.5
	-2501.3	-2317.1	-2283.6	-2202.8	-2190.4
	-2501.4	-2317.6	-2267.9	-2203.9	-2189.5
mean	-2501.1	-2317.4	-2270.4	-2201.8	-2199.4
standard deviation	0.295	0.207	4.975	0.994	6.938

K-Plot

mean		183.7	47.0	68.6	2.4
L'(K)		183.7	47.0	68.6	2.4
L''(K)		136.7	21.6	66.2	2.4
ΔK	0	661.748	4.342	66.611	0.349

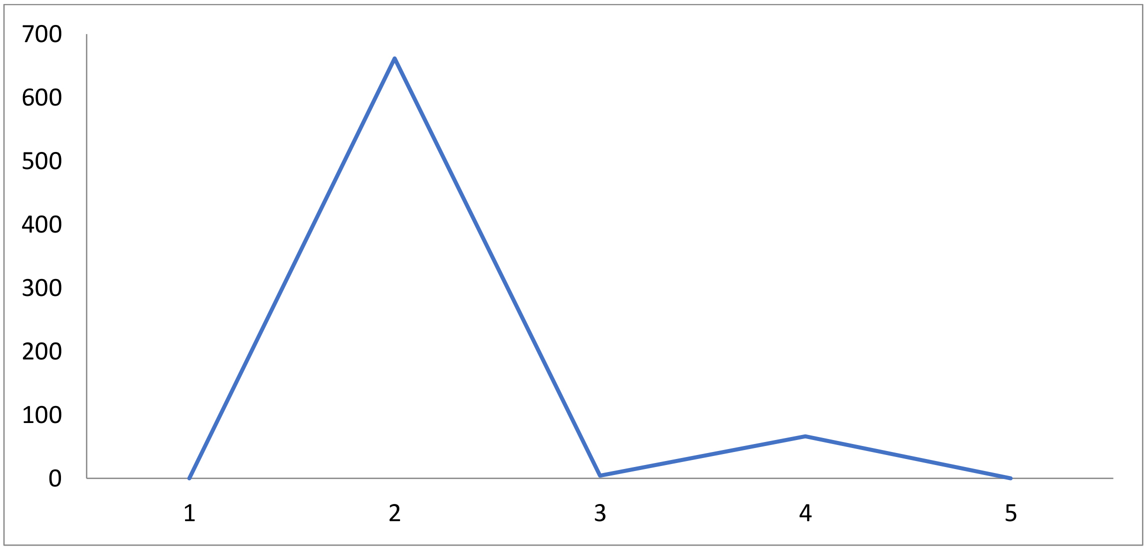

Supplement: Supplementary file 1 — Additional file 1: Table S1. Primers used in the study. Table S2. Microsatellite analysis. 2a. Genetic diversity. 2b. Complete Microsatellite data set. Table S3. Results of the Bayesian inference for the 119 samples collected from the three zones. [file 12879_2021_6120_MOESM1_ESM.pdf]
